# Succession changes of fermentation parameters, nutrient components and bacterial community of sorghum stalk silage

**DOI:** 10.3389/fmicb.2022.982489

**Published:** 2022-08-04

**Authors:** Yawei Zhang, Xinyan Tao, Qingshan Liu, Yue Jiao Zhang, Jiabao Xu, Weiyu Zhang, Jing Wang, Dandan Zhang, Bo Li, Lulu Wang, Jing Cheng, Yuan Qing Zhang

**Affiliations:** ^1^College of Animal Science, Shanxi Agricultural University, Taiyuan, China; ^2^Sorghum Research Institute, Shanxi Agricultural University, Jinzhong, China

**Keywords:** bacterial community, ensiling characteristics, fermentation parameters, nutrient components, sorghum stalk

## Abstract

To better understand the ensiling characteristics of sorghum stalk, the dynamic changes of fermentation parameters, nutrient components and bacterial community of sorghum stalk silage were analyzed by intermittently sampling on day 0, 1, 3, 7, 14, 28, and 56 of ensiling duration. The results showed that high-moisture sorghum stalk was well preserved during ensiling fermentation, with the DM loss of 4.10% and the little difference between the nutrients of sorghum stalk before and after ensiling. The pH value of silage declined to its lowest value of 4.32 by Day 7 of ensiling, and other fermentation parameters kept steady since Day 28 of ensiling. The amplicon sequencing analysis revealed that the alpha diversity parameters of silage bacterial community including Shannon index, observed features, Pielou evenness and Faith PD gradually declined (*P* < 0.01) with ensiling duration. Principal coordinate analysis (PCoA) revealed that bacterial profiles of raw material would experience a succession becoming a quite different community during ensiling fermentation. Taxonomic classification revealed a total of 10 and 173 bacterial taxa at the phylum and genus level, respectively, as being detected with relative abundances higher than 0.01% and in at least half samples. LEfSe analysis revealed that 26 bacterial taxa were affected by sampling timepoint (*P* < 0.05 and LDA score > 4). When focusing on the dynamic trend of silage bacterial taxa, lactic acid bacteria successfully dominated in the bacterial community on Day 1 of ensiling, and the bacterial community almost came to a plateau by Day 28 of ensiling, with *Lactobacillus* and *Leuconostoc* as the dominant genera. In a word, the succession of fermentation parameters, nutrient components and bacterial community indicate a successful dominance establishment of LAB and a fast advent of fermentation plateau, suggesting that high-moisture sorghum stalk can be ensiled directly, but the pH of mature silage is a little high.

## Introduction

Sorghum is one kind of gramineous annual herb C4 plant, characterized as great resistance of drought, waterlogging, soil salinity and acidity, infertility, and commonly distributed in tropical, subtropical and temperate regions (Ibeto et al., 2011; Joy et al., 2021). Nowadays, sorghum grows as one of top five cereal crops with an annually global planting area and yield of 40 million hectares and 59 million tons, and China is one of the main sorghum producers (∼3.6 million tons per year) and consumers with an annual consumption around 8.4 million tons in 2020 (FAOSTAT, 2022). As sorghum plant is low in the ratio of crop to stalk (0.5–0.8), sorghum production would likely accompany with a large amount of sorghum stalk, which could be an important feedstock for industrial production or feedstuff for livestock husbandry if properly used. Indeed, it is used as a critical roughage for ruminants feeding, acting as an important energy source via microbial fermentation in the rumen. As well, primarily consisting of cellulose, hemicellulose and lignin, sorghum stalk is also considered to be an ideal raw material for bioethanol production because of its high saccharification yield (Ibeto et al., 2011). In either way, the well utilization of sorghum stalk would make the waste transferred into an economical source of raw materials, avoiding the problems of land occupancy, burning pollution or fire hazards (Zhao et al., 2019; He et al., 2020a,b).

Generally, constant supply of raw materials is a critical factor dictating production efficiency for either factory production or animal feeding. Of the alternatives, ensiling is a common way to provide a year-round supply of seasonal high moisture materials (He et al., 2020c). Moreover, more than a preservation method, ensiling is also a processing method due to the combined effects of bacteria, enzymes and organic acids, which would likely contribute to the improved efficiency of biomass utilization (Ambye-Jensen et al., 2014; He et al., 2019). But of note, preserving in a high-moisture condition would likely bear a higher risk of spoilage and significant loss relative to drying processing. The microorganisms in silage play a critical role in the fermentation process. In essence, ensiling fermentation is a competition process of lactic acid bacteria and undesirable microorganisms, thus more attention is worthily paid to the succession of bacterial community that dictates silage quality (He et al., 2020d). Monitoring the changes in the bacterial community during fermentation gives an insight into understanding and improving the ensiling process. Gallagher et al. (2018) investigated the dynamic bacterial microbiome during sweet sorghum ensiling, results showed that sweet sorghum silage fermentation involves a shift in the lactic acid bacterial profile from *Leuconostocaceae, Lactococcus, Fructobacillus*, and *Weissella* to *Lactobacillus*. More recently, Wang et al. (2022) conducted a survey of bacterial community compositions during the ensiling of whole-plant sorghum and revealed the differences during the ensiling duration not only for the distinct bacterial community but also for specific functional metabolites. Moreover, it is recommended not to use until the silage fermentation reaches to its plateau, which could assure the constant components supply in raw materials. To the present, much research has evaluated the nutritional value of sorghum stalk and its silage as well as the effects of kinds of processing methods (Foster et al., 2019; Dos Santos et al., 2021; Puntillo et al., 2022), but little literature makes efforts to study the dynamic changes of sorghum stalk silage, especially bacterial community.

Thus, this study was conducted to investigate the dynamic changes of fermentation parameters, nutrient components and bacterial community of sorghum stalk silage by intermittently sampling on day 0, 1, 3, 7, 14, 28, and 56 of ensiling fermentation. Expecting a diverse epiphytic microbiome at packing and a rapidly changing silage environment, we explore for the first time the bacterial community successions of sorghum stalk silage. The results would throw light on the dynamic rule of sorghum stalk silage, thus providing guideline of silage improvement and utilization.

## Materials and methods

### Raw material preparation and experiment design

Fresh sorghum stalk (Jinnuo No.3) was manually harvested on the trial field of Sorghum Research Institute of Shanxi Agricultural University on October 17, 2021. Specifically, sorghum plant were randomly collected in 5 quadrats (2 m × 3 m) and then were cut into ∼2 cm length using a straw breaker (KJ-400, Kunjieyucheng Machinery Equipment Co., Beijing, China) after the ears removed. The prepared sorghum stalk deriving from each quadrat was separately packed and compacted (759.7 ± 10.1 kg/m^3^) into a laboratory silage bucket in seven duplicates, which were dedicated to the sequential sampling on day 0, 1, 3, 7, 14, 28, and 56 of ensiling duration. The buckets have a capacity of five liters and are fitted with threaded caps for post-compacted sealing. In total, 35 buckets of sorghum stalk silage (5 replicates × 7 sampling timepoints) were prepared and kept at ambient temperature (15–25°C), and then sampled for the analyses of nutrient composition, organic acids, plate count and bacterial community. The weight of each bucket was recorded both after and before being sealed and opened for calculating dry matter (DM) losses.

### Fermentation parameter analysis

The buckets were opened in a clean bench, and a sample of 30 g silage was immersed in 270 ml distilled water and kept at 4°C refrigerator overnight, and then the slurry was filtered with qualitative filter paper. The filtrate was dedicated to the determination of pH value, lactate, formate, acetate, propionate and butyrate, in which pH value was recorded by digital pH meter (PHS-3C, INESA Scientific Instrument Co., Ltd., Shanghai, China), and organic acids were simultaneously analyzed using high performance liquid chromatography (HPLC, 1260 Infinity II, Agilent Technologies, Waldbronn, Germany) equipped with Hi-Plex H column (300 mm × 7.7 mm) and G7114A UV-detector under the running conditions of oven temperature 60°C, mobile phase 5 mmol/l H_2_SO_4_, flow rate 0.7 ml/min and injection volume 20 μl. The concentration of ammoniacal nitrogen in filtrate was determined with phenol-sodium hypochlorite colorimetry described by Broderick and Kang (1980).

For microbial plate count, another 30 g sample was extracted with 270 ml of sterile saline and then the supernatant was diluted in ten folds. The lactic acid bacteria (LAB) and coliform bacteria were incubated using Lactobacilli De Man Rogosa Sharpe (MRS) agar (G-CLONE Biotechnology Co., LTD, Beijing, China) and Violet Red Bile (VRB) agar (G-CLONE Biotechnology Co., LTD, Beijing, China) at 30°C and then the well diluted plate (30∼300 cfu) was counted after 2–3 days incubation. Yeast and molds were separately enumerated on Rose Bengal agar (G-CLONE Biotechnology Co., Ltd., Beijing, China) following a 3-day incubation at 28°C.

### Nutrient composition analysis

The remaining silage was dried in a forced-air oven at 65°C for 48 h to determine dry matter content and then ground (1 mm screen) for chemical analysis. According to the procedures of Association of Official Agricultural Chemists (AOAC International, 2000), neutral detergent fiber (NDF) and acid detergent fiber (ADF) were analyzed using an A220 Fiber Analyzer (ANKOM Technology, Macedon, NY, United States) without the use of heat-stable amylase, and the results were expressed inclusive of residual ash. Nitrogen content was measured using an automatic Kjeldahl apparatus (Kjeltec 8400, Foss Analytics, Hillerød, Denmark) and crude protein (CP) was calculated by Nitrogen (N) × 6.25. Ether extract (EE) was extracted by an extraction system (ANKOM XT10, ANKOM Technology, Macedon, NY, United States). Ash was measured by the 4-h combustion in a muffle furnace at 550°C following sufficient carbonization. Gross energy (GE) was determined using an oxygen bomb calorimeter (ZDHW-8, Automatic calorimeter, Hebi, China). Water soluble carbohydrates (WSC) contents of raw sorghum stalk were measured by the reducing sugar assay using a spectrophotometer (T2602, Yoke Instrument Co., Ltd., Shanghai, China).

### Bacterial amplicon sequencing and data processing

Referring to the method of Yu and Morrison (2004), silage whole genomic DNA was extracted with E.Z.N.A. Soil DNA Kit (Omega Bio-tek, Norcross, GA, United States) following the instructions. The purity and concentration of the extracted DNA were determined by NanoDrop 2000 (NanoDrop Technology, Wilmington, DE, United States), and then agarose gel electrophoresis was conducted to verify the integrity of the DNA. Subsequently, the verified DNA was amplified using the primer pairs (338F: ACTCCTACGGGAGGCAGCAG; 806R: GGACTACHVGGGTWTCTAAT) targeting at the V3-V4 regions of bacterial 16S rDNA gene, where the amplification conditions were as followings: pre-denaturation at 95°C for 3 min, followed by 27 temperature control cycles (denaturation at 95°C for 30 s, then annealing at 55°C for 30 s, and extension at 72°C for 45 s) and stable extension at 72°C for 10 min. Meanwhile, the PCR reaction system (20 μl) consisted of 5 × FastPfu Buffer 4 μl, 2.5 mM dNTPs 2 μl, FastPfu Polymerase 0.4 μl, BSA 0.2 μl, F/R primers (2.5 μmol/l) 0.8 μl, DNA template 10 ng and ddH_2_O. Each sample was amplified in triplicate and the three amplicons were mixed together for the following enrichment and purification. Amplicons concentration was quantitated by real-time qPCR (Quantus™,Promega Corporation, United States), and agarose gel electrophoresis was used to detect their molecular weight and integrity. Sequencing library was established by the use of NEXTflex™ rapid DNA sequencing kit (Bioo Scientific, United States) according to the merchandise instructions, and then was sequenced on Illumina MiSeq PE300 (Illumina Inc., San Diego, CA, United States) generating 300 bp length paired reads.

Off-line sequencing data were processed and analyzed using the platform of QIIME2 (version 2021.08) as described previously with modification (Bolyen et al., 2019; Zhang et al., 2020). Briefly, the barcode and primer sequences were removed from the raw paired reads by the trim-paired method integrated in the q2-cutadapt plugin (Martin, 2011). The generated paired reads were denoised, merged, dereplicated and chimera-filtered by the denoise-paired method integrated in the q2-dada2 plugin to obtain amplicon sequence variants (ASVs) and their frequency distribution tables (Callahan et al., 2016). This process was achieved by truncating forward and reverse reads at position 270 and 200 bases, respectively. Taxonomic classification was performed using the classify-sklearn command (Pedregosa et al., 2011) of the q2-feature-classifier plugin with a pretrained naïve Bayesian classifier. The classifier was pretrained on the sequences of targeted region of 16S rRNA gene and corresponding taxonomy extracted from Silva 138 SSU database (Robeson et al., 2021) using the fit-classifier-Naïve-Bayes method from the q2-feature-classifier plugin.

Next, to analyze phylogenetic diversity (PD), a phylogenetic tree was generated using the align-to-tree-mafft-fasttree pipeline integrated in the q2-phylogeny plugin. To assess if sample richness has been fully observed, alpha rarefaction plots were generated based on the Shannon index and Faith PD metrics using the alpha-rarefaction visualizer of the q2-diversity plugin. To comparably analyze the bacterial diversity among samples, the sequence count of all samples was standardized by rarefying them to the same number of sequences (the smallest sampling size, 37,960 in this study) using the rarefy command of the q2-feature-table plugin. The rarefied feature table and the phylogenetic tree were then used to compute alpha diversity indices, including the Shannon index, observed features, Faith PD, and Pielou evenness and beta diversity metrics, including unweighted and weighted UniFrac distance, using the core-metrics-phylogenetic pipeline integrated in the q2-diversity plugin.

### Statistical analysis

The effects of sampling timepoints on fermentation parameters and nutrient components were evaluated using one-way analysis of variance in the GLM procedure of SAS 9.4 (SAS Institute Inc., Cary, NC, United States) with a general linear model as follows:


$$Y_{i}=\mu +T_{\text{i}}+e_{\text{i}}$$

where Y_i_ is the observation of sorghum stalk silage at the i^th^ sampling timepoints; μ and e_i_ represent the least square mean and random residual error, respectively; T_i_ is the fixed effect of ensiling duration at the i^th^ sampling timepoint. Duncan’s test was used for multiple comparisons, with difference declared significant at *P* < 0.05. For microbial diversity analysis, the differences in alpha microbial diversity among different timepoint were evaluated by Kruskal–Wallis test through alpha-group-significance command from q2-diversity plugin. PCoA was applied to visualize silage bacterial communities based on the unweighted and weighted UniFrac distances at the ASVs level using the qiime2R (version 0.99.6) package in R (version 4.1.2). Permutational multivariate analysis of variance (PERMANOVA) (Anderson, 2014) was used to test differences in beta diversity among sampling timepoints for significance using the beta-group-significance command of the q2-diversity plugin.

For microbial composition analysis, at each taxonomic level, we defined the taxa with relative abundance > 0.01% in at least one sample as identified, while those with relative abundance > 0.01% and presented in more than half samples as detected and used for downstream analysis. Bacterial composition profiles were summarized at phylum and genus levels, respectively. Relative abundance of bacterial taxa were arcsine square root transformed and then compared among different time-points using one-way analysis variance in the GLM procedure as described above. The more stringent linear discriminant analysis (LDA) effect size (LEfSe) was performed to further identify differentially abundant bacterial taxa, as described by Segata et al. (2011), and taxa with LDA score > 4 and *P* < 0.05 were considered to be significantly different.

## Results and discussion

### Dynamic changes of ensiling fermentation characteristics of sorghum stalk silage

The ensiling characteristics covering DM, DM loss, pH value, organic acids content and microbial counts of sorghum stalk silage are summarized in Table 1. As well known, ensiling is a common way to preserve high moisture forage via the help of LAB metabolism converting WSC into organic acids (mainly lactate), whereby decreasing the pH value and inhibiting the activity of undesirable microorganisms (e.g., clostridium, molds, and yeasts) (McDonald et al., 1991). Silage quality is largely affected by the characteristics of raw material such as moisture content, WSC content, buffering capacity and epiphytic microorganisms. In the present study, the DM content of fresh sorghum stalk was 23.53%, which was quite lower than the recommended DM content (30–35%) for good corn silage (Kung et al., 2018). Generally, higher moisture forage would bear higher risk of effluent loss and spoilage during ensiling process because clostridial organisms can thrive in wet conditions and convert lactic to butyric acid, which would hinder the decrease of pH value and resulting in remarkable nutrient loss (McDonald et al., 1991; Kung et al., 2018). The upside is that high moisture would be beneficial to the compaction at ensiling, especially for the tubular hollow materials like sorghum stalk or rice straw. The population of LAB, coliform bacteria, yeasts and molds were 6.97, 7.31, 5.72, 5.37 log cfu/g FM, respectively, indicating that the count of epiphytic organisms on raw sorghum stalk was quite high, which would contribute to vigorous silage fermentation. It is documented that greater than 5.0 log cfu/g FM LAB at ensiling is necessary for well-preserved silage (Lin et al., 1992), where the fast dominance establishment of acid-producing bacteria would cause a rapid pH decrease and eliminate undesirable fermentation (Pahlow et al., 2003). The weak acidity (pH 5.93) and low organic acid content (1.87, 0.63 g/kg FM lactate and formate) of raw sorghum stalk might also benefit the fermentation of these undesirable microorganisms at the early stage of ensiling fermentation. Moreover, the low WSC concentration (19.40 g/kg DM) might be an obstacle to a low enough pH decline of silage fermentation.

**TABLE 1:** Dynamic changes of dry matter, pH, organic acids, microbial counts and dry matter loss of sorghum stalk silage during ensiling process.

Item	0 d	1 d	3 d	7 d	14 d	28 d	56 d	SEM	*P*-value
Dry matter, %	23.53^a^	23.67^a^	23.44^a^	23.36^a^	23.32^a^	22.62^ab^	22.03^b^	0.149	0.01
Dry matter loss, %	–	0.00^e^	0.40^d^	1.70^c^	2.90^b^	3.60^ab^	4.10^a^	0.027	<0.01
pH	5.93^a^	5.84^a^	4.79^b^	4.32^c^	4.37^c^	4.35^c^	4.40^c^	0.117	<0.01
Ammoniacal nitrogen, g/kg FM	0.01^e^	0.05^d^	0.05^d^	0.07^c^	0.08*^bc^*	0.09^b^	0.13^a^	0.006	<0.01
Lactate, g/kg FM	1.87^e^	3.08^d^	6.36^c^	7.02^bc^	7.90^ab^	8.27^a^	8.47^a^	0.439	<0.01
Formate, g/kg FM	0.63	0.78	0.82	0.62	0.83	1.23	0.90	0.070	0.29
Acetate, g/kg FM	ND	ND	2.16^d^	3.55^c^	4.07^ab^	4.28^a^	3.77^bc^	–	–
LAB, log cfu/g FM	6.97^cd^	7.62^bc^	8.12^b^	9.05^a^	7.70^cd^	7.47^bc^	6.42^d^	0.182	<0.01
Coliform bacteria, log cfu/g FM	7.31^a^	6.42^b^	5.35^c^	5.64^bc^	<2.00	<2.00	<2.00	–	–
Yeasts, log cfu/g FM	5.72^ab^	6.53^a^	5.20^bc^	4.97^bc^	4.75^c^	3.84^ab^	3.06^ab^	0.154	<0.01
Molds, log cfu/g FM	5.37^a^	5.83^a^	3.96^b^	<2.00	<2.00	<2.00	<2.00	–	–

LAB, lactic acid bacteria; FM, fresh matter; ND, not detected; “–,” default; SEM, standard error of means; propionate and butyrate were not detected in this study. ^a–e^Means in the same row followed by different superscript letters differ (*P* < 0.05).

In terms of the ensiling characteristics, DM content slightly decreased (*P* = 0.01) during ensiling process, and DM loss and ammoniacal nitrogen concentration increased (*P* < 0.01) in the prolonged silage. It should be primarily owed to the activities of plant enzymes and microorganisms burning nutrients, mainly carbohydrate and protein, and generating metabolizable water, carbon dioxide and ammonia (Bernardes et al., 2018; Wang et al., 2022). In detail, the relative high LAB population (*P* < 0.01) and sharp decrease of coliform bacteria, yeasts (*P* < 0.01) and molds counts during ensiling process suggested a successful dominance establishment of LAB fermentation. As a consequence, silage pH was sharply lowered (*P* < 0.01) down to pH 4.32 by Day 7 of ensiling fermentation due to the accumulation of organic acids [lactate (*P* < 0.01), acetate and formate]. However, due to the limitation of fermentation substrate, the final pH of sorghum stalk silage is slightly higher than the desirable threshold pH 4.20. In a whole, the dynamic changes of these ensiling characteristics indicates that the fermentation of sorghum stalk silage would come to a relative steady status after 28 days fermentation.

### Dynamic changes of nutrient component of sorghum stalk silage

The dynamic concentrations of primary nutrients covering GE, CP, EE, NDF, ADF and ash in sorghum stalk silage are presented in Table 2. The GE of raw sorghum stalk was low with a value of 15.91 MJ/kg, likely due to its relatively high content of crude ash (9.61% DM). Meanwhile, the raw sorghum stalk had low values of CP (6.96% DM) and EE (1.96% DM), along with moderate levels of NDF (43.69% DM) and ADF (24.52% DM). As ensiling fermentation was extended, the concentrations of NDF, ADF and ash were remarkably increased (*P* < 0.01) to the values of 47.98, 32.27, and 10.24% DM by the end (day 56). Given that plant enzymes and microorganisms mainly use soluble proteins and WSC as substrates during the ensiling process, resulting in the DM loss in the way of metabolizable water, carbon dioxide, ammonia, and volatile fatty acids. On the contrary, the structural materials of plants, including fiber and minerals, would be retained to a greater extent. This DM concentration effect caused by disproportionate nutrient consumption might be the reason for the increase in concentrations of NDF, ADF, and ash as ensiling duration (He et al., 2019; Wang et al., 2022). Meanwhile, it is also suggested that the nutrient components of sorghum stalk are somewhat altered during ensiling process, which would likely exert an effect on its feeding value. In specific, the numerical values of nutrient components of sorghum stalk before and after ensiling fermentation only had a low difference in the present study, in line with the low level of dry matter loss (4.10% DM), indicating ensiling processing well preserved the nutrients of high moisture sorghum stalk.

**TABLE 2:** Dynamic changes of nutrient component of sorghum stalk silage during ensiling process.

Item	0 d	1 d	3 d	7 d	14 d	28 d	56 d	SEM	*P*-value
Gross energy, MJ/kg	15.91^cd^	15.77^cd^	15.72^d^	15.92^c^	16.19^b^	16.12^b^	16.43^a^	0.046	<0.01
Crude protein, %	6.96	6.66	6.86	6.57	7.03	7.40	7.18	0.103	0.34
Ether extract, %	1.96	2.24	2.27	1.83	2.06	1.99	1.98	0.057	0.34
NDF, %	43.69^b^	43.97^b^	43.07^b^	42.80^b^	44.92^b^	44.20^b^	47.98^a^	0.396	<0.01
ADF, %	24.52^c^	25.05^c^	24.44^c^	27.85^b^	25.58^c^	29.71^b^	32.27^a^	0.542	<0.01
Ash, %	9.61^b^	9.35^b^	9.35^b^	9.40^b^	10.09^a^	10.07^a^	10.24^a^	0.079	<0.01

NDF, neutral detergent fiber; ADF, acid detergent fiber; SEM, standard error of means. ^a–d^Means in the same row followed by different superscript letters differ (*P* < 0.05).

### Alpha and beta diversity of bacterial community in sorghum stalk silage

In the present study, a total of 1,968,149 pair-end reads were generated in the amplicon sequencing, with an average of 56,232 ± 5,427 pair-end reads per sample. For further analysis, 1,625,303 valid amplicon sequences were selected in the sequential process of primer removal, denoising, sequence consolidation and chimera removal, with an average of 46,437 ± 4,338 effective sequences per sample. Furthermore, only 1,987 representative sequences (i.e., ASVs) with an average length of 419 ± 11 bases, were harvested after repetitive sequences removed. The sequencing index Good’s coverage of each sample was over 0.999, while the rarefaction curves based on Shannon index and Faith PD for each sample level out as the sampling depth outnumbered 20,000 (Supplementary Figure 1), indicating that the sequencing analysis was deep enough to cover the entire bacterial community, assuring the representativeness of the sequencing data.

As with the alpha diversity of bacterial community, the parameters Shannon index, observed features, Pielou evenness and Faith PD gradually declined (*P* < 0.01) as ensiling fermentation was extended (Table 3). It is suggested that the diversity of bacterial community in the sorghum stalk silage decrease during ensiling process. In the early stages of ensiling fermentation, facultative and obligate anaerobic microorganisms such as Enterobacteria, Clostridia, certain bacilli and yeasts can theoretically compete with the LAB flora for the nutrients (Pahlow et al., 2003). Ensiling is a competition process of lactic acid bacteria and undesirable bacteria in essence (He et al., 2020a), where the becoming anaerobic condition would inhibit the growth of aerobic bacteria (e.g., fungi and molds) and initiate the activity of facultative anaerobe such as *Pediococcus pentosaceus*, *Enterococcus faecalis*, and *Lactobacillus plantarum*, consequently generating low pH environment to cease the fermentation (Pahlow et al., 2003). Many species of the epiphytic microbes belong to the obligate aerobic bacteria, and their growth is inhibited soon after the silo is sealed. Thus, the higher diversity of bacterial community at the early stage of ensiling fermentation should be owed to the co-existence of aerobic bacteria and facultative anaerobic bacteria, and the dominance establishment of LAB would lead to the decrease of bacterial community diversity later. Furthermore, it is inferred that LAB successfully dominate in the sorghum stalk silage, coinciding with the indication of fermentation parameters like pH value and bacterial population.

**TABLE 3:** Variation in the alpha diversity of bacterial community in sorghum stalk silage during ensiling process.

Item	0 d	1 d	3 d	7 d	14 d	28 d	56 d	SEM	*P*-value
Shannon index	6.18^a^	6.11^a^	4.91^b^	5.22^b^	4.67^b^	3.83^c^	3.29^c^	0.196	<0.01
Observed features	498^a^	462^a^	370^b^	371^b^	307^bc^	240*^cd^*	195^d^	20.5	<0.01
Pielou evenness	0.69^a^	0.69^a^	0.58^b^	0.61^b^	0.57^b^	0.48^c^	0.43^c^	0.018	<0.01
Faith PD	22.49^a^	21.21^a^	18.28^b^	17.72^b^	16.11^bc^	15.36^c^	13.10^d^	0.592	<0.01

^a–d^Means in the same row followed by different superscript letters differ (*P* < 0.05). SEM, standard error of means.

**FIGURE 1:**
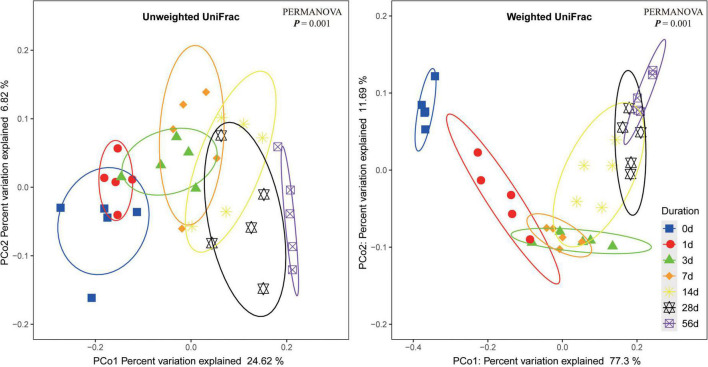
Dissimilarity of the bacterial profiles in sorghum stalk silage at different ensiling duration. Distance between samples based on similarity in ASVs composition was calculated using unweighted UniFrac (left) and weighted UniFrac distance (right), and visualized using PCoA plots. The impact of ensiling duration on the clustering pattern of bacterial communities was tested using PERMANOVA. The ovals in varied colors represent 95% confidence interval of silage bacterial profiles at different ensiling duration.

Principal coordinate analysis of bacterial community in the sorghum stalk silage based on either unweighted UniFrac or weighted UniFrac metrics are illustrated in Figure 1. The results showed that the samples on each sampling day were individually clustered within a narrow range, indicating well repeatability of the samples. The overlapping part of the clustered samples from contiguous sampling days gradually decreased as ensiling fermentation was prolonged, and the samples from non-neighboring sampling days were significantly different (PERMANOVA *P* < 0.05), suggesting that the bacterial community of raw material would experience a succession becoming a quite different community during ensiling fermentation. Such an alteration of bacterial community would be necessarily accompanied with the corresponding changes of metabolites whereby the parameters of fermentation quality varied. Additionally, not significant difference between the samples on Day 28 and Day 56 of ensiling might suggest that ensiling fermentation might have reached a steady status by Day 28.

### Relative abundance of bacterial community in sorghum stalk silage

**FIGURE 2:**
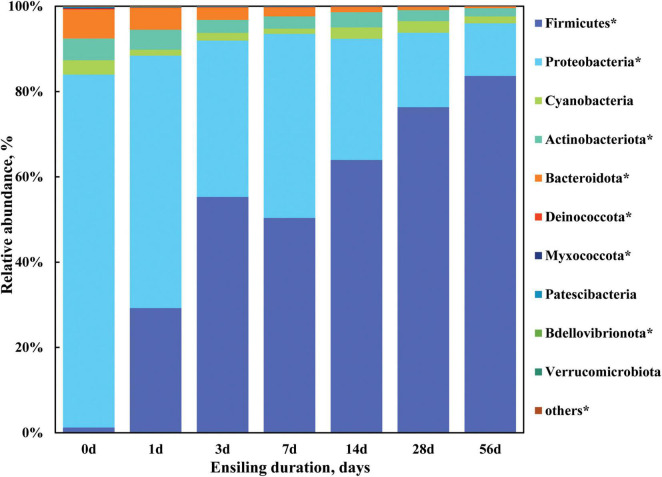
Dynamic changes of the bacterial composition at the phylum level in the sorghum stalk silage with ensiling duration. Taxa name with asterisk (*) denoted highly significant ensiling duration effect (*P* < 0.01).

To further investigate the profile of bacterial community of sorghum stalk silage, bacterial composition was analyzed on phylum level and genus level. Taxonomic profiling revealed a total of 19 bacterial taxa at the phylum level as being identified, 10 of which were classified as being detected (Figure 2) according to the cutoff defined in the section “Materials and methods.” As illustrated in Figure 2 and Supplementary Table 1, the relative abundance of detected bacterial phyla remarkably changed (*P* < 0.05) when ensiling duration extended, where *Proteobacteria*, *Bacteroidota*, and *Actinobacteriota* were the top three dominant phyla with a relative abundance of 82.76, 6.80, and 5.12% in the raw material (Day 0), respectively, which was gradually substituted by phylum *Firmicutes* (from 1.22 to 83.68%) during ensiling process, with the relative abundance decreased (*P* < 0.05) to 12.32, 0.39, and 1.89% by Day 56 of ensiling fermentation, respectively. Moreover, *Firmicutes* became the most abundant phylum of bacterial community in the sorghum stalk silage since Day 3 of ensiling. It is indicated that sorghum stalk silage fermentation involves a shift in the bacterial community from *Proteobacteria* to *Firmicutes*. Similarly, McGarvey et al. (2013) reported that the relative abundance of *Proteobacteria* in the bacterial population of alfalfa forage before and after ensiling reduced from 89.6 to 26.9%, whereas that of *Firmicutes* increased from 8.1 to 70.6% after 40 d of ensiling. Much research has revealed that the majority of the bacterial community involved in lactic acid fermentation in silage belong to the phylum *Firmicutes* (Pang et al., 2011; Ogunade et al., 2018).

Meanwhile, the individual relative abundance of *Cyanobacteria*, *Deinococcota*, *Myxococcota*, *Patescibacteria*, *Bdellovibrionota*, *Verrucomicrobiota*, and the others phyla all decreased (*P* < 0.05) in the extended silage. Generally, aerobic microorganisms consume oxygen during the early stage of ensiling and the activity of anaerobes like *Lactobacillus* leads to pH decline, inhibiting less acid-tolerant microorganisms like *Clostridium* and *Enterobacter* (Dunière et al., 2013). It is well known that the majority of the bacterial community involved in lactic acid fermentation in silage belong to the phylum *Firmicutes* (Pang et al., 2011; Ogunade et al., 2018), the dominance establishment of LAB would lead to their overwhelming abundance, and the inhibition of undesirable microorganisms should be blamed for the decreased abundance of other phyla. Additionally, it could be speculated that the fermentation of sorghum stalk silage almost came to a plateau by Day 28 of ensiling given that no difference was found on the relative abundance of bacterial community on phylum level relative to that on Day 56 (Figure 1).

**FIGURE 3:**
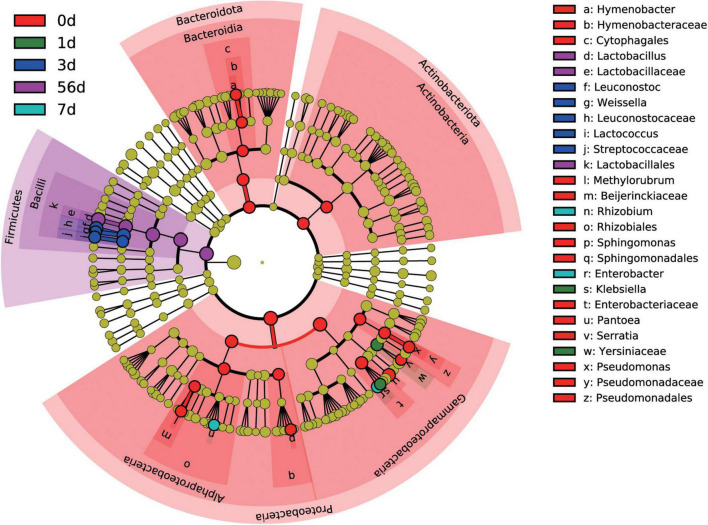
Differential silage bacterial taxa (*P* < 0.05, and LDA score > 4) among the different ensiling duration by LEfSe.

At the genus level, a total of 276 bacterial taxa were identified, of which 173 were considered as detected. To identify differentially abundant taxa among the sampling timepoint, a more stringent LEfSe analysis was performed. Results revealed that 26 bacterial taxa were affected by sampling timepoint (*P* < 0.05 and LDA score > 4), with day 0 (raw material) exhibiting high abundance of *Hymenobacter*, *Methylobacterium-Methylorubrum*, *Sphingomonas*, *Pantoea*, *Serratia*, and *Pseudomonas*, day 1 exhibiting high abundance of *Klebsiella*, day 3 exhibiting high abundance of *Leuconostoc*, *Weissella*, and *Lactococcus*, day 7 exhibiting high abundance of *Allorhizobium-Neorhizobium-Pararhizobium-Rhizobium* and *Enterobacter*, and day 56 exhibiting high abundance of *Lactobacillus*, at the genus level, respectively (Figure 3). Noticeably, all differentially abundant taxa identified by LEfSe analysis were categorized into *Proteobacteria*, *Firmicutes*, *Bacteroidota*, and *Actinobacteriota*.

**FIGURE 4:**
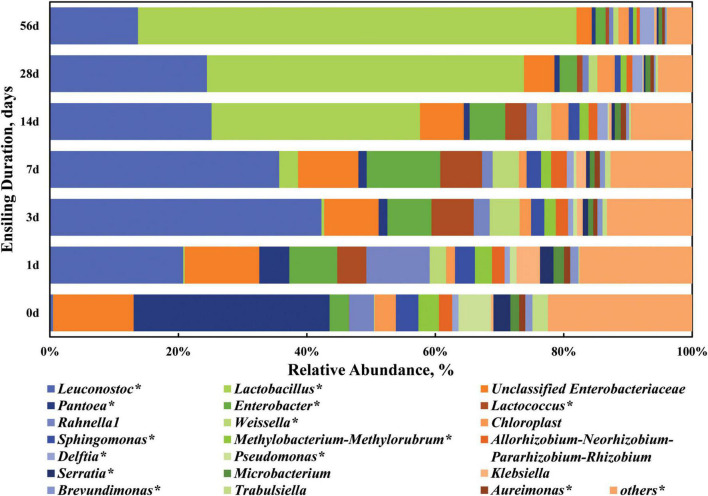
Dynamic changes of the top 20 abundant bacterial taxa at the genus level in the sorghum stalk silage with ensiling duration. Taxa name with asterisk (*) denoted highly significant ensiling duration effect (*P* < 0.01).

Thereafter, the dynamic changes of the top 20 abundant bacterial taxa at the genus level are clarified in Figure 4 and Supplementary Table 2. When focusing on the dynamic trend of each genus in a whole, the relative abundance of some genera like *Leuconostoc*, *Enterobacter*, *Lactococcus*, *Weissella*, and *Klebsiella* varied (*P* < 0.05) in a quadratic curve during ensiling process, while that of some genera like *Lactobacillus*, *Pantoea*, *Chloroplast*, *Sphingomonas*, *Methylobacterium-Methylorubrum*, *Allorhizobium-Neorhizobium-Pararhizobium-Rhizobium*, *Delftia*, *Pseudomonas*, *Serratia*, *Aureimonas*, and *Brevundimonas* experienced a linear change (*P* < 0.05). *Pantoea* and *Enterobacter* (including unclassified *Enterobacteriaceae*) were the two most abundant genera in the fresh sorghum stalk with the proportion of 30.49 and 15.56%, while there was a low abundance (0.60–4.68%) of *Pantoea* in the silage, even on Day 1 of ensiling. The genus *Pantoea* is a diverse group of non-encapsulated, non-spore-forming, yellow-pigmented, rod-shaped Gram-negative, facultative anaerobic bacteria in the *Enterobacteriaceae* family (Tambong, 2019). Many strains of *Pantoea* show striking environmental versatility and adaptability, and possess a variety of biosynthetic and biodegradative capabilities, thus epiphytic populations of *Pantoea* have been identified on many plants as well as on vegetables, fruits and grains (Walterson and Stavrinides, 2015). Ogunade et al. (2018) reported that the relative abundances of *Pantoea*, *Pseudomonas*, and *Sphingomonas* in alfalfa silage were negatively correlated with its ammonia-N concentration, which might be due to inhibition of other proteolytic bacteria like Clostridia. Enterobacteria are usually the second most numerous bacterial group of the epiphytic microflora active in the silo and thus the most important in their competition with the LAB flora, producing primarily acetic acid (Pahlow et al., 2003). Enterobacteria are also important in reducing NO_3_, resulting in the production of nitrites and nitrogen oxide gases. Therefore, the genera *Enterobacter* and unclassified *Enterobacteriaceae* are undesirable in silage in that they would compete with LAB for nutrients and produce gases, especially when WSC content is low in the raw materials.

When the stalk was ensiled, *Leuconostoc* (20.77–42.26%) and *Enterobacter* (15.32–20.83%) were the top two genera during the first 7 days of ensiling process, and later *Lactobacillus* (32.47–68.28%) and *Leuconostoc* (13.71–25.17%) became the dominant genera, indicating that sorghum stalk silage involves a shift in the bacterial community from *Pantoea* to *Leuconostoc* and finally *Lactobacillus*. Their increased abundances would expectedly promote acid production and pH decline. In general, *Enterococcus*, *Lactococcus*, and *Leuconostoc* initiate silage fermentation and create a suitable environment for the development of lactobacilli, and then those cocci are replaced by more acid-tolerant *Lactobacillus* species like *L. plantarum* and *L. brevis* (Pahlow et al., 2003). Consistently, many studies reported that the majority of the bacterial community involved in lactic acid fermentation in silage belong to the genera *Lactobacillus*, *Leuconostoc*, *Pediococcus*, *Lactococcus*, and *Weissella* (Pang et al., 2011; Yang et al., 2019). In comparison, the relative abundances of *Leuconostoc*, *Lactobacillus*, *Lactococcus*, *Weissella*, and *Delftia* in the mature sorghum stalk silage on Day 56 of ensiling was higher (*P* < 0.01) and those of *Pantoea*, *Chloroplast*, *Sphingomonas*, *Methylobacterium-Methylorubrum*, *Allorhizobium-Neorhizobium-Pararhizobium-Rhizobium*, *Pseudomonas*, *Serratia*, *Aureimonas*, and *Brevundimonas* were lower (*P* < 0.05) relative to those in the raw material. Most species of *Weissella* are obligate heterofermentative bacteria that produce lactate and acetate as major end products of sugar metabolism (Graf et al., 2016). *Weissella* species are also considered early colonizers as they are outcompeted by acid-tolerant *Lactobacillus* species due to the pH drop as fermentation progresses (Graf et al., 2016; Ogunade et al., 2018). As well, the decrease of genera *Sphingomonas*, *Methylobacterium-Methylorubrum*, and *Pseudomonas* is desirable in the silage. *Sphingomonas* is a kind of Gram-negative aerobic Alpha-Proteobacteria with extraordinary ability of degrading wide range of xenobiotic compounds and could be animal pathogens (White et al., 1996). *Methylobacterium-Methylorubrum* are aerobic, neutrophilic and facultative methylotrophic bacteria commonly found in plants, whose abundance is positive correlation with silage pH (Doronina et al., 2002; Ogunade et al., 2018). *Pseudomonas* is one kind of the most common bacteria in soil and can survive in an anaerobic environment and their presence is undesirable in silage due to its potential production of biogenic amines (Dunière et al., 2013). *Serratia* is a genus of facultative anaerobic bacteria usually involved in the production of 2,3-butanediol. Moreover, some species of *Serratia* may produce prodigiosin with antifungal properties (John Jimtha et al., 2017). From the above, the succession of bacterial community on phylum and genus level both suggest that LAB dominance is successfully established at the beginning, resulting in an advent of fermentation plateau by Day 28 of ensiling.

## Conclusion

The results of this study showed that high-moisture sorghum stalk could be well preserved during ensiling fermentation indicated by the low level of DM loss and the little difference in the nutrients of sorghum stalk before and after ensiling. As expected, bacterial succession analysis proved a successful dominance establishment of LAB and a fast advent (28 days of ensiling) of fermentation plateau. Noteworthily, although high-moisture sorghum can be ensiled, the pH of mature silage seemed a little high likely due to the lack of fermentation substrate. Dynamic changes in bacterial composition could provide some new insights into the improvement of the quality of sorghum stalk silage, for example, the development of compound lactic acid bacteria including *Leuconostoc* spp. as a specific inoculant of sorghum stalk silage.

## Data availability statement

The original contributions presented in this study are publicly available. This data can be found here: NCBI, PRJNA853895.

## Author contributions

YwZ, QL, and YQZ designed this study. XT, DZ, BL, and JC prepared the silage sample and measured the nutrient composition. XT, YJZ, JX, WZ, JW, and LW performed the fermentation parameter analysis, DNA isolation, and sequencing of library construction. YwZ, XT, and YQZ conducted the bioinformatics and statistical analyses and interpreted data. YwZ and YQZ were responsible for the manuscript writing. All authors read and approved the final manuscript.
