# Supplementary material for: Succession changes of fermentation parameters, nutrient components and bacterial community of sorghum stalk silage
Source: Front Microbiol. 2022 Aug 4;13:982489. doi: 10.3389/fmicb.2022.982489 (PMC9386229; doi:10.3389/fmicb.2022.982489)
Supplement: Supplementary file 1 [file Data_Sheet_1.docx]

Supplementary Material

| Supplementary Table 1. Relative abundance of bacterial community at the phylum level in the sorghum stalk silage, % | | | | | | | | | |
| --- | --- | --- | --- | --- | --- | --- | --- | --- | --- |
|  | Ensiling duration | | | | | | | SEM | *P* value |
| Items | 0d | 1d | 3d | 7d | 14d | 28d | 56d |  |  |
| Firmicutes | 1.22^e^ | 29.23^d^ | 55.31^c^ | 50.36^c^ | 63.96^b^ | 76.34^a^ | 83.68^a^ | 4.684 | <0.01 |
| Proteobacteria | 82.76^a^ | 59.18^b^ | 36.66^c^ | 43.18^c^ | 28.41^d^ | 17.46^e^ | 12.32^e^ | 4.048 | <0.01 |
| Cyanobacteria | 3.33^a^ | 1.40^b^ | 1.78^ab^ | 1.18^b^ | 2.68^ab^ | 2.70^ab^ | 1.64^b^ | 0.219 | <0.05 |
| Actinobacteriota | 5.12^a^ | 4.72^ab^ | 3.06^bc^ | 2.90^bc^ | 3.55^abc^ | 2.61^c^ | 1.89^c^ | 0.275 | <0.01 |
| Bacteroidota | 6.80^a^ | 5.05^b^ | 2.89^c^ | 2.16^cd^ | 1.22^cd^ | 0.71^d^ | 0.39^d^ | 0.434 | <0.01 |
| Deinococcota | 0.21^a^ | 0.12^b^ | 0.08^bc^ | 0.05^bc^ | 0.05^bc^ | 0.06^bc^ | 0.03^c^ | 0.014 | <0.01 |
| Myxococcota | 0.21^a^ | 0.03^b^ | 0.06^b^ | 0.06^b^ | 0.02^b^ | 0.02^b^ | 0.01^b^ | 0.013 | <0.01 |
| Patescibacteria | 0.15^a^ | 0.10^ab^ | 0.08^ab^ | 0.07^b^ | 0.06^b^ | 0.05^b^ | 0.03^b^ | 0.011 | 0.04 |
| Bdellovibrionota | 0.04^b^ | 0.06^a^ | 0.03^bc^ | 0.02b^cd^ | 0.01^cd^ | 0.01^cd^ | 0.01^d^ | 0.005 | <0.01 |
| Verrucomicrobiota | 0.06^a^ | 0.03^ab^ | 0.03^b^ | 0.01^b^ | 0.01^b^ | 0.02^b^ | 0.01^b^ | 0.005 | 0.02 |
| others | 0.09^a^ | 0.07^ab^ | 0.03^bc^ | 0.01^c^ | 0.02^c^ | 0.02^c^ | 0.01^c^ | 0.008 | <0.01 |
| Note: SEM, standard error of means.  ^a-e^ Means in the same row followed by different superscripts differ (*P* < 0.05). *P* values were calculated based on arcsine square root transformed relative abundances among different ensiling duration using GLM procedure in SAS. | | | | | | | | | |

| Supplementary Table 2. Relative abundance of the top 20 abundant bacterial taxa at the genus level in the sorghum stalk silage, % | | | | | | | | | |
| --- | --- | --- | --- | --- | --- | --- | --- | --- | --- |
|  | Ensiling duration | | | | | | | SEM | *P* value |
| Item | 0d | 1d | 3d | 7d | 14d | 28d | 56d |  |  |
| *Leuconostoc* | 0.48^d^ | 20.77^c^ | 42.26^a^ | 35.70^ab^ | 25.17^bc^ | 24.44^c^ | 13.71^c^ | 2.562 | <0.01 |
| *Lactobacillus* | 0.02^d^ | 0.23^d^ | 0.46^d^ | 2.95^d^ | 32.47^c^ | 49.37^b^ | 68.28^a^ | 4.674 | <0.01 |
| Unclassified *Enterobacteriaceae* | 12.54 | 11.59 | 8.46 | 9.39 | 6.81 | 4.75 | 2.36 | 1.436 | 0.49 |
| *Pantoea* | 30.49^a^ | 4.68^b^ | 1.36^b^ | 1.29^b^ | 0.90^b^ | 0.80^b^ | 0.60^b^ | 2.086 | <0.01 |
| *Enterobacter* | 3.02^bc^ | 7.45^ab^ | 6.86^ab^ | 11.44^a^ | 5.54^bc^ | 2.71^bc^ | 1.59^c^ | 0.780 | <0.01 |
| *Lactococcus* | 0.03^b^ | 4.55^ab^ | 6.59^a^ | 6.50^a^ | 3.32^ab^ | 0.83^b^ | 0.52^b^ | 0.687 | <0.01 |
| *Rahnella1* | 3.87^ab^ | 9.85^a^ | 2.47^ab^ | 1.66^ab^ | 1.65^ab^ | 0.96^b^ | 0.65^b^ | 1.068 | 0.25 |
| *Weissella* | 0.10^d^ | 2.56^b^ | 4.67^a^ | 4.10^a^ | 2.21^b^ | 1.39^bc^ | 0.81^cd^ | 0.306 | <0.01 |
| *Chloroplast* | 3.30^a^ | 1.40^b^ | 1.78^ab^ | 1.18^b^ | 2.68^ab^ | 2.70^ab^ | 1.63^b^ | 0.218 | <0.05 |
| *Sphingomonas* | 3.50^a^ | 3.10^ab^ | 2.07b^cd^ | 2.25^abc^ | 1.70^cde^ | 0.91^de^ | 0.65^e^ | 0.223 | <0.01 |
| *Methylobacterium-Methylorubrum* | 3.22^a^ | 2.66^a^ | 1.79^b^ | 1.57^bc^ | 1.42^bc^ | 0.92^cd^ | 0.54^d^ | 0.177 | <0.01 |
| *Allorhizobium-Neorhizobium-*  *Pararhizobium-Rhizobium* | 2.05^ab^ | 1.96^ab^ | 1.89^ab^ | 2.44^a^ | 1.38^abc^ | 0.91^bc^ | 0.49^c^ | 0.173 | 0.01 |
| *Delftia* | 1.00^b^ | 0.81^b^ | 0.82^b^ | 1.08^b^ | 1.59^ab^ | 1.57^ab^ | 2.25^a^ | 0.121 | <0.01 |
| *Pseudomonas* | 5.03^a^ | 1.05^b^ | 0.60^b^ | 0.40^b^ | 0.21^b^ | 0.07^b^ | 0.04^b^ | 0.350 | <0.01 |
| *Klebsiella* | 0.40^b^ | 3.65^a^ | 0.89^b^ | 1.52^ab^ | 0.41^b^ | 0.14^b^ | 0.36^b^ | 0.330 | 0.04 |
| *Serratia* | 2.65^a^ | 2.10^ab^ | 0.82^bc^ | 0.57^c^ | 0.48^c^ | 0.25^c^ | 0.28^c^ | 0.221 | <0.01 |
| *Microbacterium* | 1.37^ab^ | 1.65^a^ | 0.80^ab^ | 0.76^ab^ | 0.94^ab^ | 0.78^ab^ | 0.56^b^ | 0.120 | 0.15 |
| *Aureimonas* | 0.94^a^ | 0.95^a^ | 0.65^ab^ | 0.82^ab^ | 0.82^ab^ | 0.53^bc^ | 0.42^c^ | 0.050 | <0.01 |
| *Brevundimonas* | 1.11^a^ | 1.30^a^ | 0.79^ab^ | 0.80^ab^ | 0.46^b^ | 0.27^b^ | 0.28^b^ | 0.196 | <0.01 |
| *Trabulsiella* | 2.44 | 0.14 | 0.71 | 0.84 | 0.30 | 0.38 | 0.06 | 0.361 | 0.64 |
| others | 22.45^a^ | 17.56^ab^ | 13.26^bc^ | 12.73^bc^ | 9.55^cd^ | 5.32^de^ | 3.91^e^ | 1.212 | <0.01 |

Note: SEM, standard error of means.

^a-e^ Means in the same row followed by different superscripts differ (*P* < 0.05). *P* values were calculated based on arcsine square root transformed relative abundances among different ensiling duration using GLM procedure in SAS.


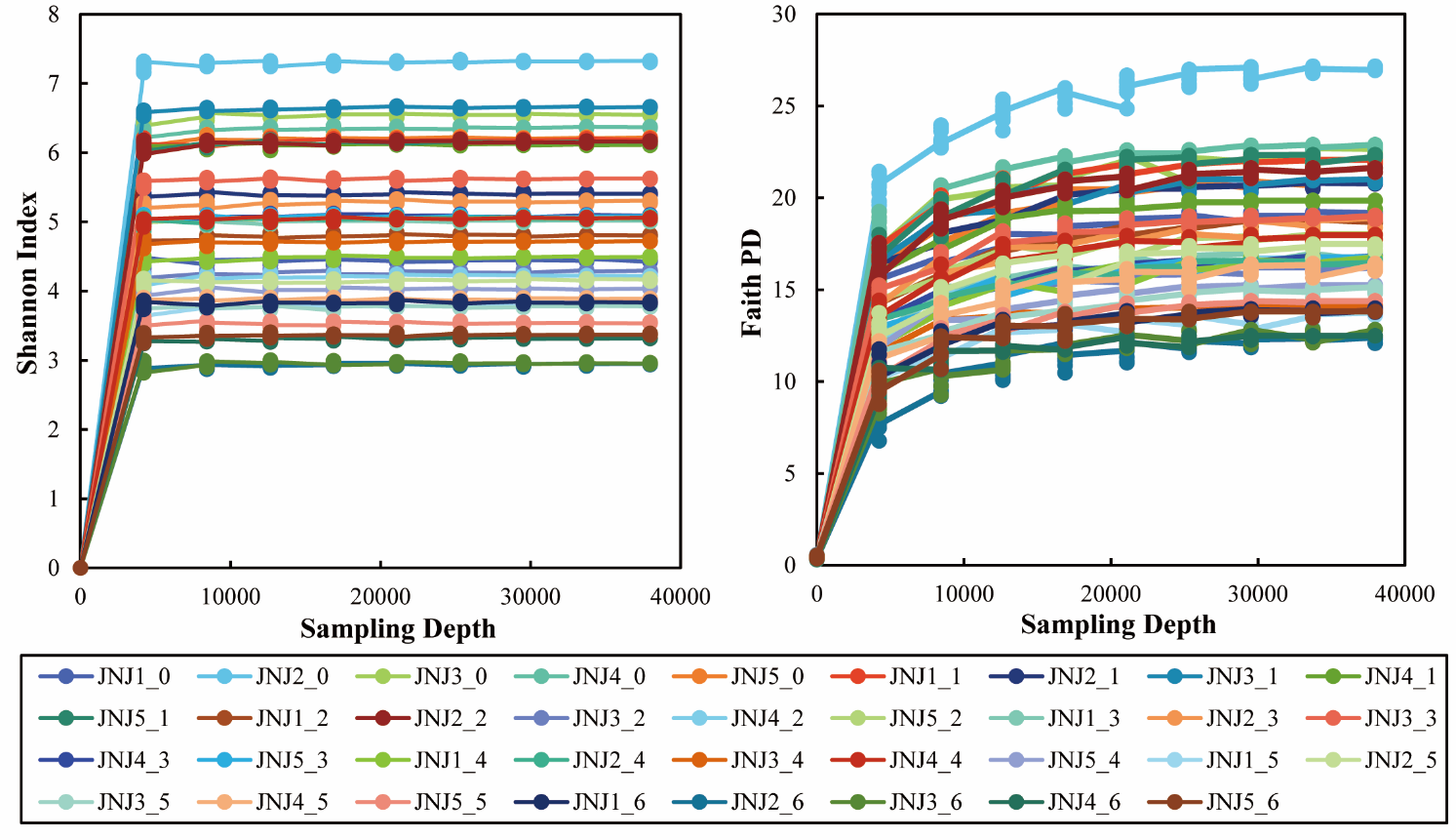


**Supplementary Figure 1.** The alpha rarefaction plots based on Shannon index (left) and Faith PD (right) for each sample.
